# Effect of Balloon Guide Catheter Utilization on the Incidence of Sub-angiographic Peripheral Emboli on High-Resolution DWI After Thrombectomy: A Prospective Observational Study

**DOI:** 10.3389/fneur.2020.00386

**Published:** 2020-05-07

**Authors:** Michael H. Schönfeld, Reza Kabiri, Helge C. Kniep, Lukas Meyer, Rosalie McDonough, Jan Sedlacik, Marielle Ernst, Gabriel Broocks, Tobias Faizy, Gerhard Schön, Bastian Cheng, Götz Thomalla, Jens Fiehler, Uta Hanning

**Affiliations:** ^1^Department of Diagnostic and Interventional Neuroradiology, University Medical Center Hamburg-Eppendorf, Hamburg, Germany; ^2^Biomedical Engineering Department, Centre for the Developing Brain, School of Biomedical Engineering & Imaging Sciences, King's College London, London, United Kingdom; ^3^Institute of Medical Biometry and Epidemiology, University Medical Center Hamburg-Eppendorf, Hamburg, Germany; ^4^Department of Neurology, University Medical Center Hamburg-Eppendorf, Hamburg, Germany

**Keywords:** ischemic stroke, cerebrovascular disease/stroke, revascularization, embolism, magnetic resonance imaging (MRI)

## Abstract

**Background:** Thrombus fragmentation causing distal emboli is a feared complication during mechanical thrombectomy (MT). We aimed to investigate the impact of procedural parameters and thrombus properties on the incidence of peripheral emboli after MT for large vessel occlusions (LVO).

**Methods:** We performed a prospective analysis of patients with LVO stroke successfully treated with MT, defined as a score of 2b, 2c, or 3 on the thrombolysis in cerebral infarction (TICI) scale. A follow-up MRI including high-resolution diffusion-weighted imaging (DWI) was performed within 24 h following MT. The primary endpoint was the number and volume of peripheral emboli, classified as punctuate DWI lesions distant to the diffusion-restricted core lesion. Further analysis included the influence of baseline characteristics, procedural and outcome parameters, and thrombus properties on peripheral emboli.

**Results:** Thirty-seven patients with successful MT met the inclusion criteria. Use of a balloon guide catheter (BGC) and TICI were the only independent predictors for a reduced number of peripheral emboli. The use of a BGC led to a significant reduction in the number and volume of peripheral emboli, with a median number/volume of peripheral emboli of 4.5/287 μl (IQR 1.25–8.25/76–569 μl) vs. 12/938 μl (IQR 4–19/242–1,836 μl). In cases where BGC was not employed, the number of peripheral emboli increased with decreasing TICI scores.

**Conclusions:** BGC-aided MT reduces the number of peripheral emboli in successful but incomplete reperfusion (TICI 2b and 2c). The effectiveness of this strategy therefore goes above and beyond that which can be demonstrated by the TICI score alone.

## Introduction

Mechanical thrombectomy (MT) has become a standard treatment for acute ischemic stroke due to large vessel occlusion (LVO) following publication of multiple randomized trials demonstrating its efficacy (1). Thrombus fragmentation, a complication of MT, can result in peripheral emboli which, in turn, can worsen clinical outcomes (2). The occurrence of peripheral emboli is not only influenced by various technical aspects of MT, but also likely by the occlusion site and individual thrombus properties (3–5). Persistent thrombi after MT can only be detected on post-interventional digital subtraction angiography (DSA) if the occluded vessel is large enough and can affect the thrombolysis in cerebral infarction (TICI) score. Additionally, higher TICI reperfusion scores are known to be associated with improved rates of favorable functional outcome (6, 7). Notably, the majority of peripheral emboli released during MT are smaller in size (8). These small fragments might cause vessel obstructions not detectable by DSA but only by magnetic resonance imaging (MRI) (9). Hence, these emboli are generally not considered in the final TICI rating after recanalization.

Recently, we found that peripheral ischemic lesions resulting from these emboli can frequently be detected on high-resolution diffusion-weighted imaging (DWI) despite supposed complete reperfusion in DSA (10). The assessment of potentially superior treatment techniques or predictors of outcome following MT by standard outcome or imaging measures requires a large patient cohort. The use of peripheral emboli as a surrogate parameter may help to tease apart the more subtle differences between the currently available treatment options.

We aimed to analyze the influence of procedural details, the location of the occlusion, as well as the thrombus properties on the occurrence of peripheral emboli, as detected by high-resolution DWI (HR-DWI). We hypothesized that the number of peripheral emboli during MT of LVO can be reduced with the utilization of balloon guide catheters (BGC).

## Materials and Methods

### Inclusion Criteria and Study Endpoints

We conducted a prospective study of patients treated with MT for LVO at a tertiary stroke center between August 2017 and March 2019. Inclusion criteria were defined as follows: (1) anterior circulation LVO; (2) thrombectomy as endovascular therapy; (3) successful reperfusion (defined as TICI ≥2b); (4) post-interventional MRI follow-up imaging including a HR-DWI sequence.

The primary study endpoint was defined as the absolute number and volume of peripheral emboli detected on HR-DWI MRI imaging following MT. A further analysis of possible factors influencing the incidence of peripheral emboli after MT was performed, including BGC utilization.

All procedures performed in studies involving human participants were in accordance with the ethical standards of the institutional and national research committee and with the 1964 Helsinki declaration and its later amendments or comparable ethical standards. The local ethics committee approved the study (Ethics Committee of the Board of Physicians Hamburg; approval number WF 019/19) and waived the requirement to obtain informed consent.

### Endovascular Treatment

If eligible, all patients received intravenous thrombolysis (IVT) with alteplase prior to MT (11). Procedures were performed via a femoral artery approach under conscious sedation or general anesthesia. The choice of thrombectomy devices, including the selection of a BGC, was at the discretion of the treating neurointerventionalist. No formal decision process was applied for the choice of treatment technique.

### Magnetic Resonance Imaging (MRI)

MRI was performed on a 1.5 T MRI scanner (Siemens Magnetom Avanto, Erlangen, Germany) within 24 h following intervention.

Axial whole-brain HR-DWI was performed using single-shot, multi-slice, spin-echo, echo-planar imaging sequences with diffusion gradients in three orthogonal directions and the following parameters: TR/TE 13000/90 ms, b-values of 0, 500 and 1,000 sec/mm^2^, matrix 192 × 192, no gap, field of view 240, slice thickness 2 mm, no gap, and an acquisition time of 457 s.

### Imaging Analysis

Density of the thrombus and the contralateral vessel was determined in 1 mm thick axial reconstructions of non-enhanced computed tomography scans (CT) by placement of 1–2 mm^2^ regions of interest. Thrombus density was represented in both Hounsfield units and as a density ratio (density of the thrombus/density of the contralateral vessel). Thrombus length was measured on multiplanar reconstructions of pre-interventional CT-angiography.

Post-interventional DSA imaging was rated according to the revised TICI definition proposed by Noser et al. and endorsed by Goyal et al. (12). Here, TICI grade 2b is defined as reperfusion of more than 50% of the expected territory, grade 2c as near-complete perfusion without a clearly visible thrombus, yet delay in contrast run-off and grade 3 as complete perfusion with physiological filling of all distal branches of the expected territory in a normal hemodynamic fashion (13).

The first pass effect (FPE) was defined as achieving near-complete reperfusion (TICI ≥2c) after single-device and adjunctive technique approach (14).

Peripheral emboli were defined as diffusion restrictions on DWI distant to a continuous core DWI lesion. They were classified as lesions either within the vascular territory of the primary LVO or emboli into new territories.

All DWI lesions were semi-automatically segmented using seed growing algorithms provided by the Analyze 11.0 software package (AnalyzeDirect, Inc., Overland Park, KS, USA). Imaging analysis was performed independently by two readers that did not participate in the endovascular procedure. Discrepancies between readers were resolved by consensus.

### Statistical Analysis

The impact of thrombus and interventional parameters on the number of peripheral emboli was first assessed in univariable regression models. Then Poisson regression was used to model the effect of independent predictors on the number of peripheral emboli. As independent parameters, we analyzed i.v. alteplase, occlusion location, thrombus density and length, devices used, number of revascularization attempts, reperfusion score, and FPE.

Data is reported using standard descriptive statistics. Wilcoxon signed rank test was performed to compare continuous variables, as appropriate. Chi Square test and Fisher's exact test were used to compare categorial variables. All statistics were calculated using SPSS 24.0 (IBM SPSS Statistics for Windows, Armonk, NY: IBM Corp.) and R 3.61 (R Core Team. R: A Language and Environment for Statistical Computing. R Foundation for Statistical Computing. Vienna, Austria, 2019). *P* < 0.05 were considered statistically significant.

## Results

### Study Population

**Figure 1:**
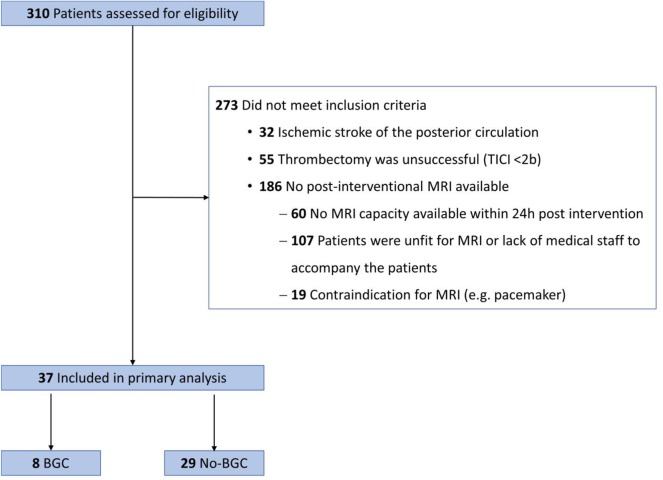
Flowchart of patients included in the study from August 2017 to March 2019. BGC, balloon guide catheter; TICI, thrombolysis in cerebral infarction; MRI, Magnetic Resonance Imaging.

Thirty-seven patients met the inclusion criteria and were prospectively enrolled in the study (Figure 1). The median age was 71 years (IQR 65.5–80) and 51.4% (19/37) were female. The median time from onset to recanalization was 275 min (IQR 227–385). On admission, the median ASPECTS was 7 (6–9) and the median NIHSS was 14 (9.5–16.5). On initial CT scans, the median thrombus length was 15 mm (IQR 10.5–20) and the median thrombus density was 60 HU (IQR 53.5–64.5). 45.9% (17/37) of all patients received i.v. alteplase prior to MT. All LVOs were located within in the anterior circulation, including 29.7% (11/37) in the terminal internal carotid artery (tICA) and 70.3% (26/37) in the M1 segment of the media cerebral artery (MCA). 18.9% (7/37) of all occlusions were tandem occlusions with concomitant extracranial ICA stenosis. Table 1 gives a detailed overview of the patient baseline characteristics and outcome data.

**Table 1:** Comparison of patient baseline characteristics, procedural and functional outcome, and study endpoints between patients treated with and without balloon guide catheters.

**Baseline characteristics**	**All patients (*n* = 37)**	**BGC (*n* = 8)**	**No-BGC (*n* = 29)**	***p*-value**
Age in years, median (IQR)	71 (65.5–80)	69.5 (64.25–80.0)	72.0 (65.5–80.0)	0.926
Female sex, *n* (%)	19 (51.4)	4 (50)	15 (51.7)	1.0
Admission NIHSS, median (IQR)	14 (9.5–16.5)	12 (7.75–16.75)	13 (9–15.5)	0.868
**Occlusion site** ***n*** **(%)**				
• Tandem occlusion	7 (18.9)	2 (25.0)	5 (17.2)	0.631
• Terminal ICA	11 (29.7)	4 (50.0)	7 (24.1)	0.203
• MCA M1	26 (70.3)	4 (50.0)	22 (75.9)	
ASPECTS, median (IQR)	7 (6–9)	7.5 (4.5–9)	7 (6–9)	0.865
Thrombus length in mm, median (IQR)	15 (10.5–20)	15.5 (12.5–24.75	15 (9.5–20)	0.459
Thrombus density in Hounsfield units, median (IQR)	60 (53.5–64.5)	57 (45–64.25)	60 (53.5–64.5)	0.518
Thrombus density ratio, median (IQR)	60 (53.5–64.5)	1.30 (1.19–1.37)	1.30 (1.15–1.50	0.555
**Procedural and functional outcome**				
i.v. Alteplase, *n* (%)	17 (45.9)	5 (62.5)	12 (41.4)	0.428
General anesthesia, *n* (%)	2 (5.4)	0 (0)	2 (6.9)	0.390
Use of a distal aspiration catheter, *n* (%)	30 (81.0)	3 (37.5)	27 (96.4)	0.002
Primary aspiration, *n* (%)	15 (40.5)	2 (25.0)	13 (44.8)	0.431
Use of a stent-retriever, *n* (%)	32 (86.5)	8 (100)	24 (82.8)	0.564
FPE, *n* (%)	11 (29.7)	3 (37.5)	8 (27.5)	0.672
Number of passes, median (IQR)	1 (1–2.5)	1.5 (1–2.5)	1.0 (1–2.75)	1.0
TICI (2b/2c/3)	16/9/12	14/6/9	2/3/3	0.450
Time symptom onset to recanalization in min, median (IQR)	275 (227–385)	338.0 (245–356)	272.5 (213.75–444.25)	0.470
NIHSS at discharge, median (IQR)	3 (1–6.5)	1 (0.25–3)	3 (0.5–6.5)	0.475
mRS 24 h, median (IQR)	3 (2.5–5)	3 (2.25–5)	3 (2.5–5)	0.79
mRS 90 d, median (IQR)	2 (1–4)	2 (2–5)	2 (1–4)	0.448
mRS 90 d 0/1/2/3/4/5/6, *n*	7/7/8/6/4/3/2	2/0/3/1/0/1/1	5/7/5/5/4/2/1	0.457
mRS 90 d ≤ 2, *n* (%)	22 (59.5)	5 (62.5)	17 (58.6%)	1.0
**Study endpoints**				
Number of peripheral emboli, median (IQR)	10 (4–15)	4.5 (1.25–8.25)	12 (4–19)	0.024
Volume core infarct in ml, median (IQR)	19.69 (8.71–46.87)	17.91 (4.85–110.67)	19.698 (8.71–42.78)	0.912
Volume of peripheral infarcts in ml, median (IQR)	0.74 (0.15–1.56)	0.29 (0.08–0.57)	0.94 (0.24–1.84)	0.034

*BGC, balloon guide catheter; ASPECTS, Alberta stroke program early CT score; ICA, internal carotid artery; FPE, first pass effect; TICI, thrombolysis in cerebral infarction; NIHSS, national institute of health stroke scale; mRS, modified Rankin scale*.

### Study Endpoints

In univariate linear regression analysis FPE, reperfusion success assessed with the TICI scale, and the use of BGC were found to be predictors for the number of peripheral emboli (Table 2).

**Table 2:** Univariate linear regression analysis of predictors for peripheral emboli.

**Parameter**	**Coefficient**	**95% CI**	***p*-value**
FPE	−7.140	−13.91 to −0.37	0.039
TICI	−7.060	−10.00 to −4.11	<0.001
BGC	−8.270	−15.75 to −0.80	0.031

*FPE, first pass effect; TICI, thrombolysis in cerebral infarction; BGC, balloon guide catheter*.

**Figure 2:**
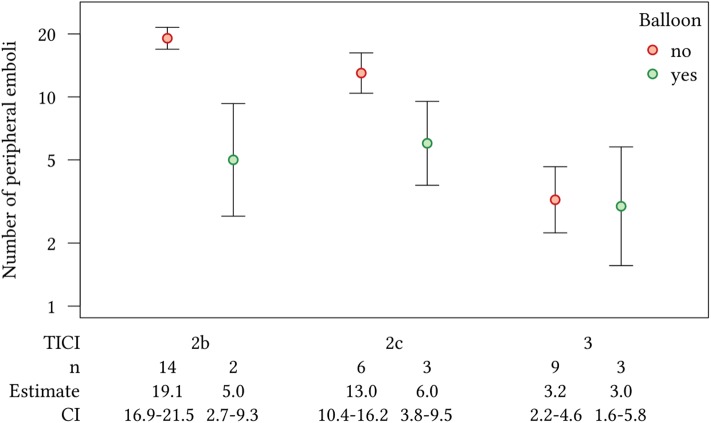
Effect plot showing the number of peripheral emboli with 95% CI in the Poisson regression model, with the interaction of TICI score and the use of a BGC.

FPE was excluded from the Poisson regression model due to its direct relationship with the TICI rating; all cases with FPE are rated as either TICI 2c or 3. Based on an ordinal scale, TICI entered the model in the subcategories 2b, 2c, and 3. The predictive value of BGC use and TICI for the number of peripheral emboli was confirmed in the Poisson regression model after adjusting for the interaction between TICI and BGC. In cases where BGC was not employed, the number of peripheral emboli decreased with increasing TICI scores. With BGC, however, the reduction of the number of peripheral emboli with increasing TICI scores was less pronounced and the number of peripheral emboli after successful but incomplete reperfusion (TICI 2b and 2c) was lower compared to patients treated without BGC (Figure 2, Table 3).

**Table 3:** Poisson regression model with interaction of TICI and the use of a BGC for the prediction of peripheral emboli.

**Parameter**	**Incidence-rate ratio**	**95% CI**	***p*-value**
(Intercept)	19.07	16.87–21.45	<0.001
TICI 2c	0.68	0.53–0.87	0.003
TICI 3	0.17	0.11–0.24	<0.001
BGC	0.26	0.13–0.47	<0.001
TICI 2c × BGC	1.76	0.79–4.11	0.173
TICI 3 × BGC	3.55	1.31–9.53	0.011

*TICI, thrombolysis in cerebral infarction; BGC, balloon guide catheter*.

**Figure 3:**
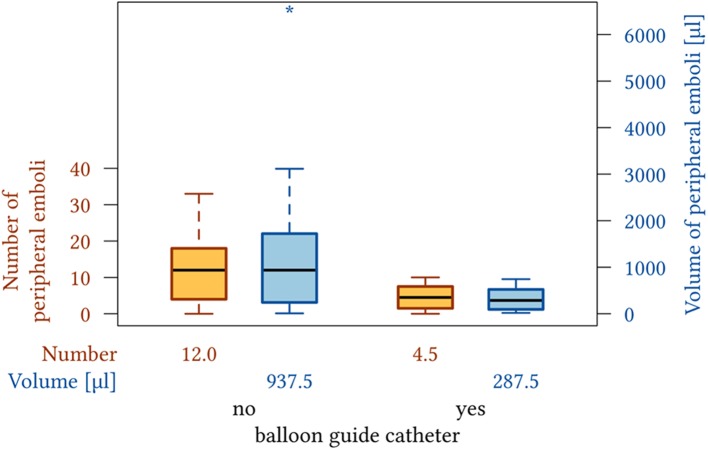
Box plot showing the number and volume of peripheral emboli seen after MT without and with the use of a BCG.

For all patients receiving BGC-aided MT, the incidence of peripheral emboli was significantly lower (median 4.5 vs. 12; *p* = 0.024), and the total volume of peripheral ischemias was significantly lower (median 938 vs. 287 μl; *p* = 0.034) compared to those who were treated without BGC use (Figure 3). The “BGC cohort” and the “non-BGC” cohort did not differ significantly regarding demographics, pre-interventional ASPECTS, occlusion location, previous administration of i.v. alteplase, thrombus properties, MT success, and total number of revascularization attempts. Distal aspiration catheters were used more often in cases where BGC was not employed (Table 1).

## Discussion

Our analysis further supports the hypothesis that the utilization of BGCs reduces the number of peripheral emboli detected as peripheral ischemia after MT of LVO. While there was no significant difference in the number of peripheral emboli when complete reperfusion was achieved (TICI 3), the use of BGC reduced the number of peripheral emboli after successful but incomplete reperfusion (TICI 2b and 2c).

This is the first study investigating the impact of baseline characteristics, procedural and outcome parameters, as well as thrombus properties on the incidence of peripheral emboli after MT with high-resolution DWI.

*In-vitro* and animal models have demonstrated the reduction in the number of peripheral emboli with BGC via two mechanisms; both antegrade flow arrest and concurrent aspiration at the balloon guide reduce migration of the entrapped thrombus or fragmented clots extracted by the stent retriever (4, 15). An *in-vitro* vascular phantom occlusion model showed that BGC offered a two-fold improvement in soft elastic clot fragmentation compared with a standard guide catheter (16). In a porcine model, Nikoubashman et al. demonstrated that BGC utilization provided reliable flow arrest and flow reversal with manual syringe aspiration, whereas aspiration with a conventional 8 F guide catheter resulted in either collapse of the vessel or oscillatory flow, potentially permitting antegrade flow (4).

The first clinical evidence for the use of BGC came from an analysis of the NASA registry data that found improved clinical outcomes and reperfusion scores (17). A meta-analysis of non-randomized trials suggested that use of BGC is associated with superior clinical and angiographic outcomes (18). Recently, analysis of the data gathered in the STRATIS Stroke Thrombectomy Registry and TRACK registry came to the same conclusion that the use of BGC is an independent predictor of clinical and angiographic outcome (19, 20). Our study further reaffirms the reperfusion benefit of BGC, beyond angiographic findings.

In MT without BGC, reperfusion success was a predictor for the number of peripheral emboli in our study. This is in line with previous studies in which reperfusion success was found to be the most important modifiable predictor for outcome (21). Accordingly, the largest proportion of patients with excellent functional outcome (mRS ≤ 1) is seen in cases in which complete reperfusion (TICI 3) was achieved (21, 22). Consequently, the neurointerventionalist performing MT should aim to achieve the best reperfusion success possible.

FPE was also a predictor for the number of peripheral emboli in the univariate analysis. Recently, Nikoubashman et al. reported that FPE was an independent factor for favorable outcome, however their analysis did not test for the influence of BGC (23). It has been shown that the use of BGC facilitates FPE and, by definition, the FPE influences the TICI score (20). It is therefore not an independent variable and was removed from the multivariate model in our study.

None of the other baseline characteristics, thrombus properties, or procedural details had a significant effect on the number of peripheral emboli in our study.

Utilizing the technique that achieves the highest possible grade of reperfusion success at first pass is important to minimize the risk of clot fragmentation and downstream emboli. Our study reinforces the benefit of BGC in achieving better reperfusion results, which in turn leads to improved clinical outcomes in patients with LVO stroke.

The clinical impact of peripheral emboli is uncertain. They may, along with other modifiable factors that are associated with poor outcomes following stroke (procedural emboli into the anterior cerebral artery territory, hypertension, hyperglycemia, and fever), help elucidate why some patients despite successful reperfusion and small core infarcts do not regain functional independency (24, 25). In our study, the total volume of peripheral emboli made up only a small fraction of the ischemic core volume. However, not only the size of the infarct core, but also infarct location, can have a significant impact on clinical outcome (26). If located within eloquent brain regions (e.g., the motor cortex or corticospinal tract) even small lesions could have a substantial impact on a patient's level of functional independence. Furthermore, primarily subclinical DWI lesions could manifest as early neurocognitive changes (27).

Our findings demonstrate the use of imaging markers for the design of future stroke studies. Results from *in-vitro* testing and porcine *in vivo* models have provided some evidence for recommendations for procedural details that were otherwise based on hypothetical considerations, (4, 16). But designing a clinical trial to provide evidence for these recommendations will be difficult. A clinical trial seeking to test a true improvement in functional outcomes to this already very effective therapeutic strategy would require many patients, as well as an extended study period. The use of a surrogate outcome measure may therefore be justified to investigate new interventions in proof-of-concept studies. Although the TICI scale is an established predictive tool, it is not able to capture fine subtleties between the current treatment options, potentially explaining the varied outcomes observed for the same reperfusion scores. Techniques that reduce the number of peripheral emboli may therefore be preferable, as they could increase the frequency of favorable outcome, thereby partially resolving such discrepancies. The authors of a recent systematic review and meta-analysis concluded that DWI could be used as surrogate outcome measure for procedural stroke after carotid revascularization (28). For acute stroke treatment, DWI infarct growth has already been proposed as a surrogate endpoint for future stroke therapy trials in order to reduce sample sizes (29). Acquiring HR-DWI to detect peripheral emboli may add to this metric.

A major limitation of this study is that we did not acquire pre-interventional HR-DWI to detect the peri-interventional occurrence of ischemic lesions. Although desirable, unnecessarily delaying treatment by nearly 8 min (the acquisition time of HR-DWI) is not justifiable. Furthermore, small ischemic lesions likely would not yet be detectable due to the acute time window (30). Some ischemias might have resulted from pre-interventional fragmentation of the thrombus. Comparing pre- and post-treatment MRI of a large cohort of patients with LVO, Gratz et al. found that patients more commonly present with a single thrombus rather than with multiple thrombi (2). Therefore, persisting vessel occlusion after recanalization of the initial LVO is likely the result of thrombus fragmentation during the MT procedure rather than the presence of a second, pre-existing thrombus. Furthermore, the reduction of emboli with the use of a BGC is in itself another convincing argument for the peri-interventional incidence of most of the peripheral emboli.

Another limitation is the low rate of BGC cases. This is due to the fact that the favored technique in our department includes the use of a large bore distal aspiration catheter, incompatible with our current BGC. Nevertheless, despite our small cohort size, the association between BGC use and reduced number of emboli is evident.

## Conclusion

Incidence of peripheral emboli after MT for LVO stroke is lower with the utilization of a BGC for successful but incomplete reperfusion (TICI scale 2b and 2c). When observed in HR-DWI this effect goes above and beyond the TICI scale. Therefore, MT with proximal flow-arrest could have a greater influence on procedural and functional outcomes than initially expected. Further studies investigating the role of BGC in MT are urgently needed to confirm these findings.

## Data Availability Statement

The raw data supporting the conclusions of this article will be made available by the authors, without undue reservation, to any qualified researcher.

## Ethics Statement

The studies involving human participants were reviewed and approved by Ethics Committee of the Board of Physicians Hamburg. Written informed consent for participation was not required for this study in accordance with the national legislation and the institutional requirements.

## Author Contributions

MS, JS, JF, and UH conceptualized the study. MS, RK, HK, JS, ME, GB, TF, BC, and GT collected the data. MS, HK, and GS performed the statistical analysis. MS, RM, LM, and UH drafted the manuscript. MS, RK, HK, LM, RM, JS, ME, GB, TF, BC, JF, and GT revised the manuscript.

## Conflict of Interest

JF: Consultant for Acandis, Boehringer Ingelheim, Codman, Microvention, Sequent, Stryker. Speaker for Bayer Healthcare, Bracco, Covidien/ev3, Penumbra, Philips, Siemens. Grants from Bundesministeriums für Wirtschaft und Energie (BMWi), Bundesministerium für Bildung und Forschung (BMBF), Deutsche Forschungsgemeinschaft (DFG), European Union (EU), Covidien, Stryker (THRILL study), Microvention (ERASER study), Philips. GT: Received personal fees as consultant/lecturer from Acandis, Bayer, Boehringer Ingelheim, Bristol-Myers Squibb/Pfizer, Daichi Sankyo, Stryker, and research grants from Bayer, Federal Ministry for Economic Affairs and Energy (BMWi), Corona-Foundation, German Research Foundation (DFG), Else Kröner-Fresenius Foundation, European Union (Horizon 2020), German Innovation Fund. The remaining authors declare that the research was conducted in the absence of any commercial or financial relationships that could be construed as a potential conflict of interest.
